# Safety programme reduces ICU mortality

**DOI:** 10.1186/cc9913

**Published:** 2011-03-11

**Authors:** KD Rooney, R Sundaram, L Gibson, RJ Price

**Affiliations:** 1 Royal Alexandra Hospital, Glasgow, UK

## Introduction

One in 10 patients admitted to Scottish hospitals are unintentionally harmed and around 50% of these events could have been avoided if lessons from previous incidents had been learned. A National Audit Office report estimated that patient safety incidents cost the NHS an estimated £2 billion a year.

## Methods

We identified a minimum of eight main elements that we should concentrate on in order to produce reliable critical care. They included VAP, CVC insertion and maintenance, peripheral vascular catheter maintenance, daily goals, multidisciplinary ward rounds, hand hygiene, and glycaemic control.

## Results

**Figure 1:**
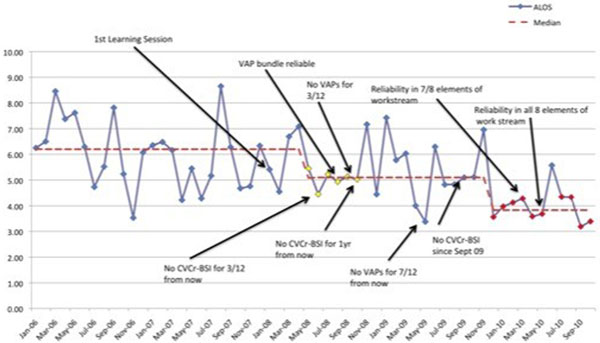
**Average ICU length of stay**.

**Figure 2:**
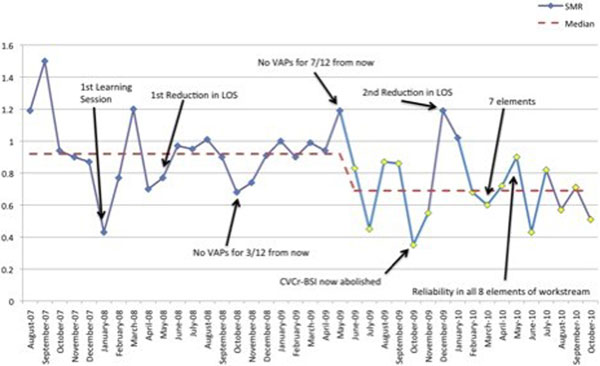
**Standardised mortality ratio**.

We have seen significant reductions in our VAP and Cr-BSI rates with more than 230 days and 440 days between events achieved, respectively. Despite an increase in the complexity and severity of cases in the last year due to Pandemic H1N1 2009, our average length of stay (Figure 1) has still reduced by 2.4 days with a 0.23 reduction in our standardised mortality ratio (Figure 2) from 0.92 to 0.69.

## Conclusions

The public display of our infection rates has helped change the culture in our ICU to one of transparency and safety. Multiple small-scale tests of change are integral to changing practice in a high-risk environment. Bundles of care, daily goals and checklists all help produce high-quality reliable healthcare.
